# Removal of Aflatoxin B_1_ Using Alfalfa Leaves as an Adsorbent Material: A Comparison between Two In Vitro Experimental Models

**DOI:** 10.3390/toxins15100604

**Published:** 2023-10-08

**Authors:** María de Jesús Nava-Ramírez, Alma Vázquez-Durán, Juan de Dios Figueroa-Cárdenas, Daniel Hernández-Patlán, Bruno Solís-Cruz, Guillermo Téllez-Isaías, Carlos López-Coello, Abraham Méndez-Albores

**Affiliations:** 1 Unidad de Investigación Multidisciplinaria (UIM) L14 (Alimentos, Micotoxinas y Micotoxicosis), Facultad de Estudios Superiores Cuautitlán (FES-C), Universidad Nacional Autónoma de México (UNAM), Cuautitlán Izcalli 54714, Mexico; mari_551293@comunidad.unam.mx (M.d.J.N.-R.); almavazquez@comunidad.unam.mx (A.V.-D.); 2 Cinvestav-IPN Unidad de Querétaro, Libramiento Norponiente No. 2000, Fraccionamiento Real de Juriquilla, Queretaro 76230, Mexico; jfigueroa@cinvestav.mx; 3 UIM L5, FES-C, UNAM, Mexico City 54714, Mexico; danielpatlan@comunidad.unam.mx (D.H.-P.); bruno_sc@comunidad.unam.mx (B.S.-C.); 4 Division of Agriculture, Department of Poultry Science, University of Arkansas, Fayetteville, AR 72701, USA; gtellez@uark.edu; 5 Departamento de Medicina y Zootecnia de Aves, Facultad de Medicina Veterinaria y Zootecnia, UNAM, Mexico City 04510, Mexico; coelloca@unam.mx

**Keywords:** aflatoxin B_1_, alfalfa leaves, adsorption, in vitro digestion models, characterization

## Abstract

An adsorbent material derived from alfalfa leaves was prepared and further characterized, and its efficacy for removing aflatoxin B_1_ (AFB_1_) was investigated. Characterization consisted of the use of attenuated total reflectance-Fourier transform infrared spectroscopy (ATR-FTIR), environmental scanning electron microscopy (ESEM), X-ray fluorescence spectroscopy (XRF), X-ray diffraction (XRD), point of zero charge (pH_pzc_), zeta potential (ζ-potential), UV-Vis diffuse reflectance spectroscopy, and spectral analysis. To determine the adsorption capacity against AFB_1_ (250 ng AFB_1_/mL), pH-dependent and avian intestinal in vitro models were used. The adsorbent inclusion percentage was 0.5% (*w*/*w*). In general, the pH-dependent model gave adsorption percentages of 98.2%, 99.9%, and 98.2%, evaluated at pH values of 2, 5, and 7, respectively. However, when the avian intestinal model was used, it was observed that the adsorption percentage of AFB_1_ significantly decreased (88.8%). Based on the characterization results, it is proposed that electrostatic, non-electrostatic, and the formation of chlorophyll-AFB_1_ complexes were the main mechanisms for AFB_1_ adsorption. From these results, it can be concluded that the adsorbent derived from alfalfa leaves could be used as an effective material for removing AFB_1_ in in vitro digestion models that mimic the physiological reality.

## 1. Introduction

Mycotoxins are toxic secondary metabolites synthesized by several species of filamentous fungi [1]. Until now, more than 300 types of mycotoxins have been known [2], including aflatoxin B_1_ (AFB_1_), which is considered one of the most toxic substances because of its highly carcinogenic potential to humans and animals [3,4]. The International Agency for Research on Cancer has classified aflatoxin as a group 1 carcinogen [5]. In general, AFB_1_ is considered a natural contaminant of various agricultural products intended for feed preparation, and the consumption of these contaminated products causes serious health problems; therefore, there are considerable economic losses in the poultry industry due to aflatoxin consumption [6,7].

To reduce the negative effects produced by aflatoxins, various strategies have been proposed; one of the most promising and used in the feed industry is the addition of adsorbent materials [8,9]. Adsorbents are compounds that are characterized by having a large molecular weight; consequently, AFB_1_ present in the contaminated feed is capable of binding to these materials without dissociating throughout the gastrointestinal tract (GIT) of the bird, limiting AFB_1_ absorption and promoting its elimination via the feces [8,9]. The most widely used mycotoxin adsorbents are inorganics such as zeolites, aluminosilicates, hydrated calcium and sodium aluminosilicate, and clays; however, they are mostly non-biodegradable compounds, and some of them release toxic components such as heavy metals and dioxins, in addition to indiscriminately adsorbing some nutrients from the diet [8,10]. In recent years, various plant-based adsorbents have been developed, which offer an efficient, economical, and environmentally friendly alternative to remove AFB_1_, in addition to maintaining the nutritional value of the diet [11]. In the scientific literature, some of the agrisorbents that have been already tested to remove AFB_1_ are based on grape and olive (pomaces, seeds, and stems), banana peel, *Formosa firethorn* (leaves and berries), lignins, micronized fibers, *Aloe vera*, lettuce, field horsetail, kale, durian peel, blueberry pomace, artichoke wastes, and almond hulls [12,13].

Due to the integral benefits that characterize a plant-based adsorbent, more research is required to evaluate the adsorption potential against AFB_1_ of other materials that are destined to be consumed with the feed. Alfalfa (*Medicago sativa* L.) is a forage used worldwide in animal feed due to its adaptability, high protein content, and low production cost [14]. The values of crude protein, crude fiber, crude cellulose, and metabolizable energy contained in alfalfa are 17.5%, 24.1%, 20%, and 1200 kcal/kg, respectively [14,15]. Little is known about the effects of fresh forage consumption by poultry [16]. For instance, Suwignyo et al. [17] reported that fresh alfalfa supplementation in ducks affected feed intake, body weight gain, and feed conversion ratio. However, it has been reported that alfalfa meal can be used in poultry diets due to its high nutritional and pigment content, lower amount of cellulose, and higher digestibility [14]. Other authors have reported that the inclusion of powdered alfalfa in poultry diets resulted in positive effects such as a reduction in the feed conversion ratio, mortality, abdominal fat, and cholesterol content of the yolk. In addition, powdered alfalfa increased the content of the pectoral muscle and body weight as well as improved the height of the villi and depth of the duodenal crypts [18,19]. In general, there is a considerable variation in the recommended levels of inclusion of alfalfa in poultry diets. Suwignyo et al. [17], Shahsavari et al. [20], and Suwignyo et al. [18] recommended an inclusion limit of 6%, 5%, and 3% (*w*/*w*), respectively. However, alfalfa has a high nutritional value as it is a good source of proteins, minerals, vitamins, flavonoids, and isoflavones [18]. In addition, alfalfa is a natural source of pigments such as xanthophylls, chlorophylls, and carotenoids, giving poultry carcasses a desirable yellowish color [15,18]. In this research, we hypothesize that the use of alfalfa as an adsorbent material could provide certain advantages in the poultry industry, since alfalfa can have a dual-purpose role, that is, as an AFB_1_ adsorbent and as a feed supplement due to its large amount of nutrients and phytochemicals. To the best of our knowledge, there are no studies on the use of alfalfa leaves as an AFB_1_ adsorbent; therefore, the aim of the present study was to prepare and characterize an adsorbent material derived from alfalfa leaves and investigate its AFB_1_ adsorption capacity in two experimental in vitro models.

## 2. Results and Discussion

### 2.1. Adsorption Experiments

#### 2.1.1. The pH-Dependent Model

**Figure 1:**
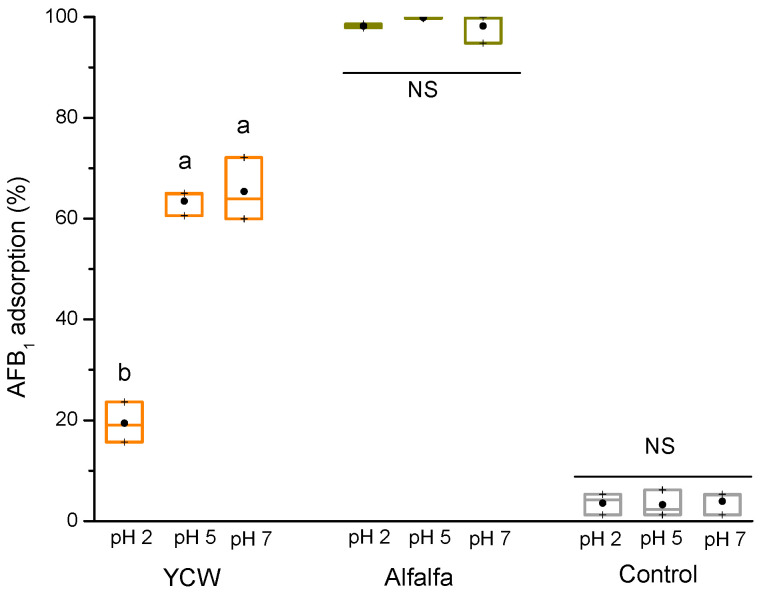
The AFB_1_ adsorption capacity of the adsorbent material derived from alfalfa leaves and the yeast cell wall (YCW) using a pH-dependent in vitro model. Boxes and whiskers not sharing a common superscript differ significantly (Tukey test *p* < 0.05). NS = not significant.

The pH is an important factor in adsorption experiments; in most cases, pH can affect the surface charge of the functional groups of the adsorbent, as well as the ionization of the toxin [21]. For this reason, an in vitro study was carried out with three different pH values (2, 5, and 7) to evaluate the adsorption capacity of the plant-based adsorbent and the YCW (reference material), using a 0.5% (*w*/*v*) inclusion rate. Figure 1 shows that the pH variation had no influence on the removal of AFB_1_ by the alfalfa adsorbent; consequently, there was no statistical significance in the percentage of AFB_1_ adsorption at the three tested pH values. In general, the percentage of adsorption of AFB_1_ with the adsorbent derived from alfalfa leaves was significantly higher at the three pH values compared to the YCW. At pH values of 2, 5, and 7, the adsorbent prepared from alfalfa leaves removed 98.2 ± 0.4%, 99.9 ± 0.2%, and 98.2 ± 2.9% of the toxin, respectively. On the contrary, the YCW adsorbed 17.4 ± 3.9%, 63.5 ± 2.5%, and 65.4 ± 6.2% of the mycotoxin, respectively. Therefore, the adsorbent prepared from alfalfa leaves removed 80.2%, 36.5%, and 33.5% more AFB_1_ than YCW at pH 2, 5, and 7, respectively. The control (without the addition of adsorbent) showed a considerable deficiency of AFB_1_ uptake (<5%).

In general, the charge of the AFB_1_ molecule depends on its acid dissociation constant (pKa = 17.79); thus, AFB_1_ is neither protonated nor deprotonated within the pH range of this research. Consequently, the variation in the pH of the medium did not affect the adsorption of AFB_1_ regardless of the surface charge of the adsorbent. These findings are in accordance with the results of other authors [11,22,23,24,25].

#### 2.1.2. The Avian Intestinal Model

**Figure 2:**
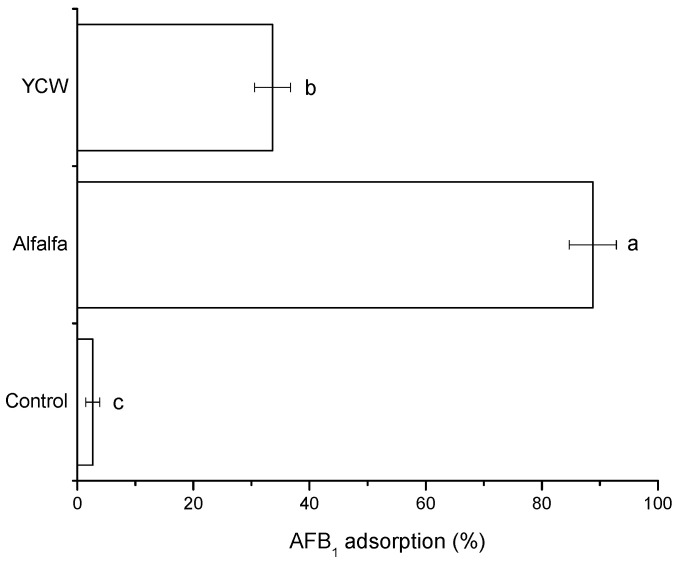
The AFB_1_ adsorption capacity of the adsorbent material derived from alfalfa leaves and the yeast cell wall (YCW) using the avian intestinal model. ^a,b,c^ Mean values ± standard error. Means not sharing a common superscript differ significantly (Tukey *p* < 0.05).

Another in vitro digestive model was also used, which aimed to simulate the conditions of the GIT of birds. Figure 2 shows the percentage of AFB_1_ adsorption. In the intestinal section, the adsorbent derived from alfalfa leaves had a significantly higher percentage of adsorption (88.8 ± 4.1%) compared to the YCW (33.6 ± 3.1%). On the contrary, the control group (without adsorbent addition) had a marked lack of AFB_1_ adsorption (<3%). Various authors have performed AFB_1_ adsorption studies under the simulation of some GIT conditions; for example, Adunphatcharaphon et al. [26] carried out a study in a standardized digestion model, which included the phases of oral, gastric, and small intestine digestion. The authors using acid-treated durian peel showed that the adsorbent had 98.4% aflatoxin uptake. Moreover, Vázquez-Durán et al. [27] carried out an in vitro digestive model simulating the GIT conditions of birds. In the study, adsorbents prepared from kale and lettuce removed 93.6% and 83.7% of the mycotoxin, respectively. In the present investigation, a clear decrease in the adsorption capacity of AFB_1_ was observed with the adsorbent derived from alfalfa leaves in the avian intestinal model compared to the pH-dependent model. The adsorption of AFB_1_ in the avian intestinal model was mainly affected by the lack of interaction between the adsorbent and the AFB_1_ because the feed matrix did not allow the encounter between the adsorbent and the adsorbate, in addition to the effect exerted by the gastric enzymes. In this context, Rasheed et al. [13] compared the efficacy of blueberry pomace (BB) to remove AFB_1_ using a static in vitro model and a model that simulated gastric conditions. The authors reported that the in vitro model at pH 3 had better adsorption capacity compared to the model that simulated gastric conditions. This decrease in the BB adsorbent efficiency was attributed to the difficulty of trapping the AFB_1_ molecule due to the presence of pepsin in the model that simulated the gastric fluid. These results are consistent with our findings. 

### 2.2. Characterization 

#### 2.2.1. FTIR-ATR

**Figure 3:**
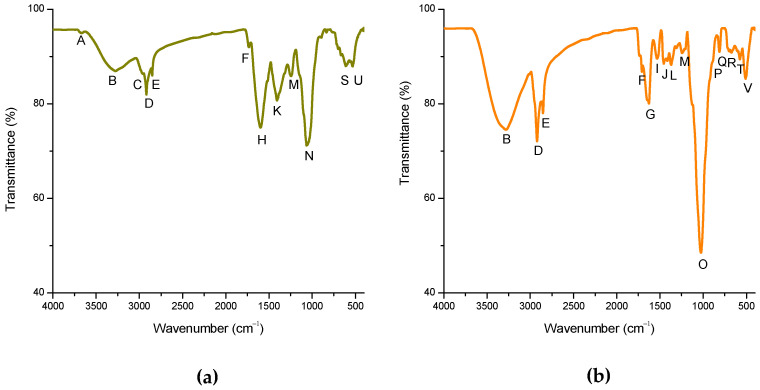
Fourier transform infrared spectroscopy (FTIR) spectrum of (**a**) the adsorbent material derived from alfalfa leaves and (**b**) the yeast cell wall (YCW).

The FTIR-ATR spectra of the adsorbent material derived from alfalfa leaves and the YCW were collected in the spectral range of 4000 to 400 cm^−1^ (Figure 3). Table 1 compiles the primary active FTIR vibrations and their functional groups. In general, the functional groups present in the adsorbent materials were further analyzed to investigate the possible interactions between the AFB_1_ molecule and the functional groups [28]. The adsorbent derived from alfalfa leaves (Figure 3a) exhibited a high intensity of the hydroxyl group at 3668 and 3280 cm^−1^, the methyl group at 2964, 2917, and 2850 cm^−1^, the vibration associated with the carboxyl group at 1599 cm^−1^, the strong vibration of aromatic compounds at 1408 and 1242 cm^−1^, and the strong molecular vibration of the C–O bond at approximately 1066 cm^−1^ [13,27,29,30,31]. On the other hand, the main bands related to chlorophylls are the C–O stretching, the C–C stretching vibration, and the bands originated from the stretching of the methyl groups [32,33]. As can be seen, both adsorbent materials mainly contained four functional groups: hydroxyl at 3281 cm^−1^, aliphatic chains at 2923 and 2853 cm^−1^, aromatic compounds at 1532, 1455, 1369, and 1244 cm^−1^, and the carbonyl group at approximately 1025 cm^−1^. Furthermore, in Figure 3b, it is also possible to distinguish three characteristic regions of the YCW adsorbent, which correspond to polysaccharides (1182–842 cm^−1^), proteins (1573–1701 cm^−1^), and lipids (2797–2990 cm^−1^) [34].

**Figure 4:**
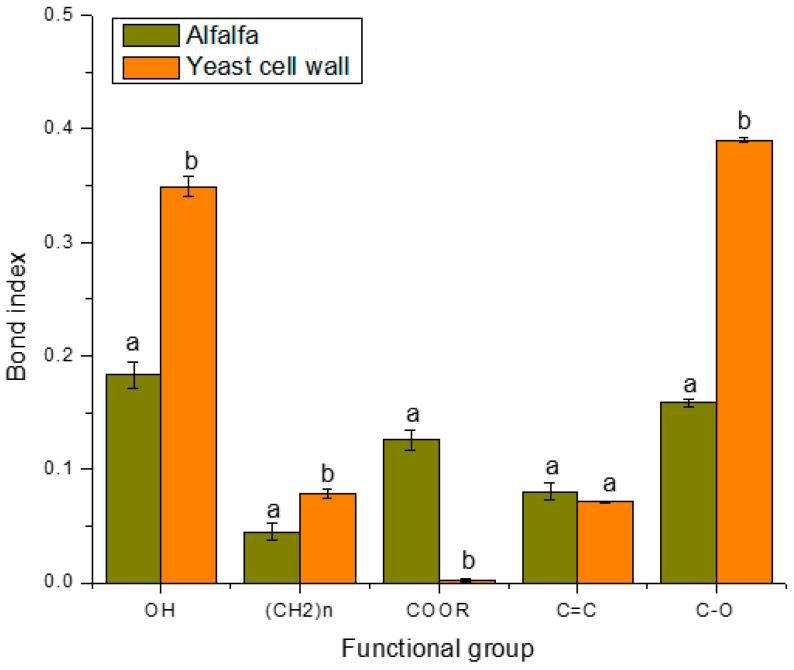
Bond indexes of the main functional groups of the adsorbent materials. ^a,b,c^ Mean values ± standard error. Means not sharing a common superscript differ significantly (Tukey *p* < 0.05).

According to what was reported by Vázquez-Durán et al. [27] and Shar et al. [29], the functional groups present in the adsorbents (hydroxyl, methyl, carboxyl, aromatic, and carbonyl) contribute to the adsorption of AFB_1_. Bearing this in mind and that the alfalfa and YCW share most of the vibrations of these functional groups, the calculation of the bond indexes was carried out to know the real amount of each functional group. According to the results (Figure 4), the adsorbent prepared from alfalfa leaves contained fewer methyl groups compared to the YCW; however, in terms of aromatic groups, there was no statistical significance between the two tested materials. On the other hand, the YCW contained 1.9 and 2.5 times more hydroxyl and carbonyl groups, compared to the adsorbent derived from alfalfa leaves. It has been reported that the adsorption of AFB_1_ can occur due to the hydrophobicity of the adsorbents, and this hydrophobicity is conferred to its surface by the presence of hydrophobic groups such as methyl and aromatics; on the contrary, if hydrophilic groups are present (hydroxyl, carboxyl, and carbonyl), the adsorption efficiency of the adsorbents is compromised [27]. It is important to note that the spectrum of the YCW lacks carboxyl groups compared to the adsorbent derived from alfalfa leaves, which shows a prominent band at around 1599 cm^−1^. It is well known that functional groups deprotonate if they are at a pH higher than their pKa [35]. In this context, the pKa of the carboxyl group is ~4.5; thus, from pH values above 4.5 the carboxyl loses the hydrogen atom; consequently, this functional group is only capable of forming hydrogen bonds with the oxygen atoms of the AFB_1_ molecule at pH of 2 (the proventriculus environment). Furthermore, the pKa of the hydroxyl group is ~11.6, the amine group is ~40, and the amide group is ~18; therefore, the presence of these three functional groups favors the formation of hydrogen bonds with the oxygen atoms of the AFB_1_ molecule in the three simulated sections (crop, proventriculus, and intestine). Regarding the YCW, several authors have reported that β-d-glucans, glucomannans, and mannan-oligosaccharides are the main components responsible for the mycotoxin adsorption [36].

#### 2.2.2. ESEM

**Figure 5:**
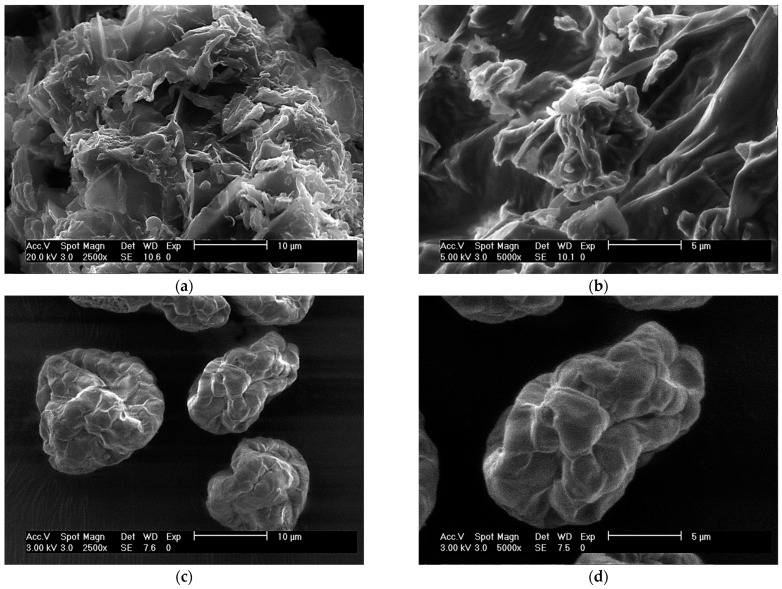
ESEM micrographs of (**a**,**b**) the adsorbent material derived from alfalfa leaves and (**c**,**d**) the yeast cell wall (YCW) at 2500× and 5000×, respectively.

The microstructure and surface morphology of both adsorbent materials were scrutinized by ESEM (Figure 5). In general, the adsorbent derived from alfalfa leaves showed a rough microstructure with large edges in the form of small sheets or ridges (Figure 5a,b). This set of microstructures could play an important role during the adsorption of AFB_1_. Shar et al. [29] suggest that functional groups and the heterogeneous microstructure on the surface of the adsorbents contribute to the uptake of mycotoxins. On the other hand, in the YCW, the microstructure of individual cells is clearly observed, with an assembly similar to a raspberry [37], ellipsoid to oval in shape with a smooth surface and some invaginations (Figure 5c,d). Hernández-Ramírez et al. [34], reported that the microstructure of the YCW is characterized by its oval shape and smooth surface with aggregates of different sizes. Unlike the alfalfa leaf-derived adsorbent, YCW has a notably less rough surface and a less-exposed area, particularities that suggest that YCW would have a lower AFB_1_ adsorption capacity.

#### 2.2.3. XRF

**Figure 6:**
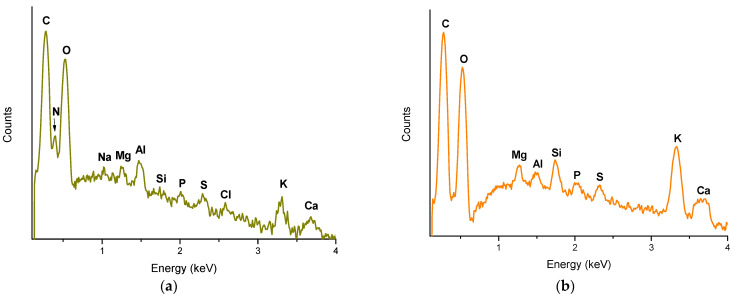
Micro X-ray fluorescence spectra of (**a**) the adsorbent derived from alfalfa leaves and (**b**) the yeast cell wall (YCW).

With the micro X-ray fluorescence technique on ESEM, it was possible to perform the micro-elemental analysis of both adsorbents. Figure 6 shows the XRF spectra of the adsorbent derived from alfalfa leaves and the YCW. The elemental analysis of the adsorbent prepared from alfalfa leaves showed a significant amount of carbon (49.44%), nitrogen (5.48%), oxygen (43.92%), and traces of sodium (0.22%), magnesium (0.17%), aluminum (0.27%), silicon (0.03%), phosphorus (0.04%), sulfur (0.07%), chlorine (0.07%), potassium (0.18%), and calcium (0.11%). In this context, Zavala-Franco et al. [10] reported that the main elements of the organic adsorbents they studied (banana peel, *Pyracantha* leaves, and *Aloe* powder) were carbon and oxygen, which is consistent with our results. On the other hand, in the XRF spectra of the YCW it can be seen that YCW has fewer elements; however, there was a significant amount of carbon (45.32%), oxygen (38.02%), potassium (7.87%), and traces of magnesium (1.32%), aluminum (0.50%), silicon (1.30%), phosphorus (0.49%), sulfur (0.81%), and calcium (1.38%). In this regard, Chen et al. [38] showed that the scanning electron micrograph of the *Cinnamomum camphora* leaf powder (CLP) adsorbent was modified in terms of the change in intensity of some peaks after adsorption; these changes in the intensity of the XRF peaks suggested that certain elements present in the adsorbent were capable of effectively removing the pollutant [39]. Thus, the adsorbent material derived from alfalfa leaves would have a better AFB_1_ adsorption capacity due to the large amount of carbon and oxygen (up to 93.36%).

#### 2.2.4. XRD

**Figure 7:**
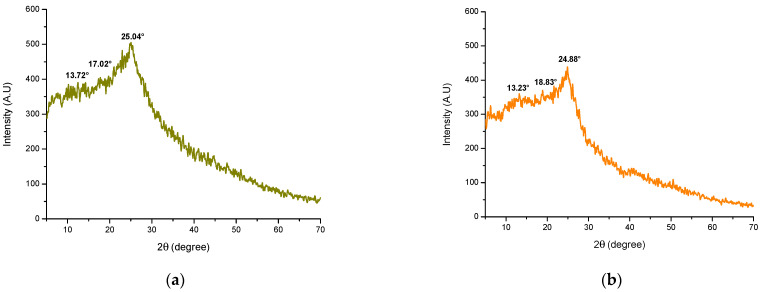
X-ray diffraction patterns of (**a**) the adsorbent derived from alfalfa leaves and (**b**) the yeast cell wall (YCW).

Figure 7 shows the X-ray diffraction pattern of the adsorbent material derived from alfalfa leaves and the YCW. In the diffractogram of the adsorbent prepared from alfalfa leaves (Figure 7a), a diffraction peak with a considerable intensity was observed at 2θ = 25.04°. Wada et al. [40] associates this peak with the presence of crystalline cellulose. Two other peaks of minor intensity at 2θ = 13.72° and 17.02° appeared, which were also related to the presence of the cellulose type I crystalline structure [41] and semicrystalline starch [10]. On the other hand, the diffractogram of the YCW (Figure 7b) showed three diffraction peaks, the most intense at 24.88° 2θ and two less intense at 13.23° and 18.83° 2θ. These peaks correspond to the polymeric crystalline structure of β-glucans [42,43]. The XRD results agree with what was found in the corresponding FTIR spectra of the adsorbent materials. For instance, in the FTIR spectrum of the adsorbent derived from alfalfa leaves, the band around 1408 cm^−1^ was associated with the presence of cellulose, and in the spectrum of the YCW, the bands located at 887, 812, 670, 575, and 508 cm^−1^ were associated with the presence of β-glucans.

#### 2.2.5. pH_pzc_ and ζ-Potential

**Figure 8:**
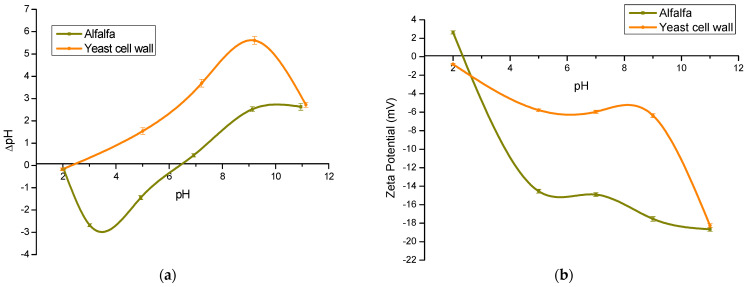
Point of zero charge (**a**) and Zeta potential (**b**) of the adsorbent materials.

The pH_pzc_ is a technique to characterize the surface charge of an adsorbent material. It is well known that the adsorption capacity depends on the variation of the pH and the degree to which the adsorbent and the adsorbate are protonated or deprotonated [44]. Figure 8a shows the pH_pzc_ of the adsorbent materials. The pH_pzc_ values of the adsorbent prepared from alfalfa leaves and YCW were 6.51 and 2.45, respectively. Rosas-Castor et al. [21] reported that the pH_pzc_ of the alfalfa adsorbent was 6.9, and Hernández-Ramírez et al. [34] reported that the pH_pzc_ of the YCW was 3.09. According to these results, the adsorbent derived from alfalfa leaves would have a better adsorption capacity at pH 2 and 5, that is, in the first two compartments of the GIT of birds, since the surface charge of the adsorbent at these pH values remained positively charged, which favors the interaction with the oxygen atoms of the AFB_1_ molecule. However, in the pH-dependent in vitro experiment, there was no statistically significant difference in the adsorption of AFB_1_ at the three pH values evaluated; thus, it can be hypothesized that at pH 7 electrostatic interaction is not the main adsorption mechanism. These results agree with Adunphatcharaphon et al. [26], who evaluated the effect of pH on AFB_1_ adsorption using durian peel.

On the other hand, ζ-potential is a technique that allows knowing the electric field originated by the surface charges of a certain material [45,46]. In this research, ζ-potential was determined in a pH range from 2 to 11 (Figure 8b). In general, the ζ-potential of the two tested adsorbents was more negative as the pH increased. Electronegative values of ζ-potential indicate a large accumulation of positive charges near the particle surface. On the contrary, electropositive ζ-potential values allow the accumulation of negative charges near the particle surface [46]. Thus, the ζ-potential of the YCW was negative in the entire pH scale that involves the GIT: pH 2 (−0.81 mV), pH 5 (−5.77 mV), and pH 7 (−5.95 mV). On the contrary, the ζ-potential of the adsorbent derived from alfalfa leaves was positive at pH 2 (+2.63 mV), and it was significantly more negative than the YCW in two of the three GIT compartments (pH 5 and pH 7), attaining ζ-potential values of −14.55 mV and −14.91 mV, respectively. Therefore, it is proposed that there is a significant contribution to the AFB_1_ adsorption when using the alfalfa leaf-derived adsorbent compared to the reference material at acidic pH values (pH 2), due to electrostatic interactions between the adsorbent and the mycotoxin [47]. 

#### 2.2.6. Determination of Chlorophylls and Carotenoids 

##### Spectral Reflectance Measurements

**Figure 9:**
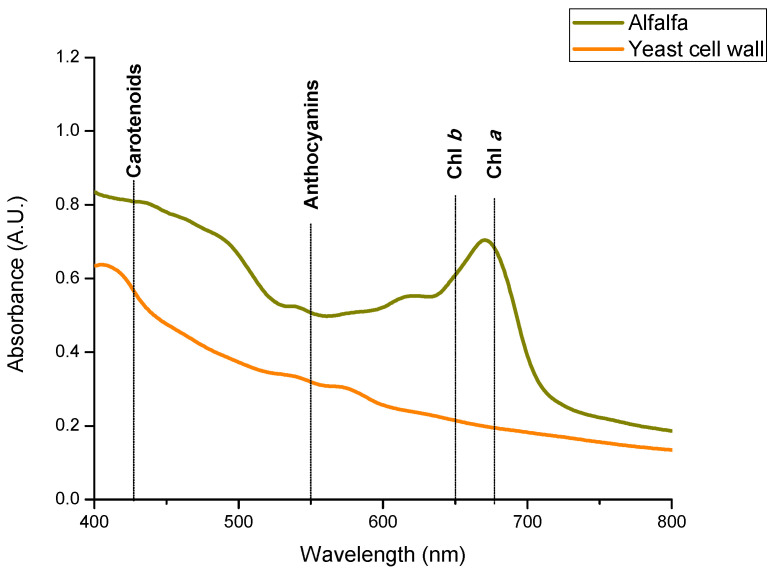
Diffuse reflectance UV-Vis spectra of the adsorbent materials.

To evaluate the presence of pigments in the adsorbent materials, the UV-Vis diffuse reflectance spectroscopy technique was utilized [12]. Figure 9 shows the diffuse reflectance spectra of the adsorbent derived from alfalfa leaves and the reference material (YCW). The adsorbent prepared from alfalfa leaves showed two representative bands (678 nm and 650 nm), which correspond to Chl *a* and Chl *b*, respectively. Moreover, the absorbance of anthocyanins is associated with the maxima at 550 nm [27]. Finally, in the range from 430 to 530 nm, the presence of carotenoids was observed [48]. These results agree with Merzlyak et al. [49], who studied the diffuse reflectance spectra of five different apple peels. The authors reported the presence of chlorophylls *a* and *b*, carotenoids, and anthocyanins. On the contrary, in the YCW, no representative bands were found (Figure 9). Nava-Ramirez et al. [31] conducted a study to test the efficacy of three adsorbents with a high chlorophyll content (lettuce, *pyracantha*, and horsetail) for AFB_1_ adsorption; the researchers demonstrated that the more chlorophylls an adsorbent has, the greater the adsorption capacity. The authors concluded that chlorophylls were able to form strong non-covalent complexes with the AFB_1_ molecule. In this research, the adsorbent derived from alfalfa leaves contained large amounts of chlorophylls; therefore, it had higher possibilities to form chlorophyll–AFB_1_ complexes. This ability to form chlorophyll–AFB_1_ complexes could explain the great adsorption potential of the adsorbent derived from alfalfa leaves.

##### Quantitative Determination of Chlorophylls and Carotenoids

To perform the quantification of pigments in both adsorbent materials, Chl *a*, Chl *b,* and total carotenoids were extracted with 96% ethanol and subsequently the absorbance of each pigment was determined spectrophotometrically. The spectrum of the adsorbent derived from alfalfa leaves (Figure S1) shows a narrow maximum band in the blue region (near 432 nm) and another band in the red region (near 665 nm). These bands correspond to the presence of Chl *a* [27,33] [27,33]. Carotenoids have a broad absorption with three shoulders within the blue region between 400 and 500 nm [50]. To know the real content of the main photosynthetic pigments in the alfalfa adsorbent, the specific absorption coefficients of Lichtenthaler and Wellburn [51] were used (Table 2). It was observed that the total chlorophyll content of the adsorbent prepared from alfalfa leaves (2759.1 ± 180.2 µg/g dry weight) was significantly higher than the total carotenoid content (16.3 ± 94.5 µg/g dry weight). The chlorophyll content in alfalfa agrees with that reported by Dziwulska-Hunek et al. [52]. Figure S1 also shows the UV-Vis spectrum of the YCW ethanolic extract; it is clearly observed that this adsorbent does not contain pigments. 

**Figure 10:**
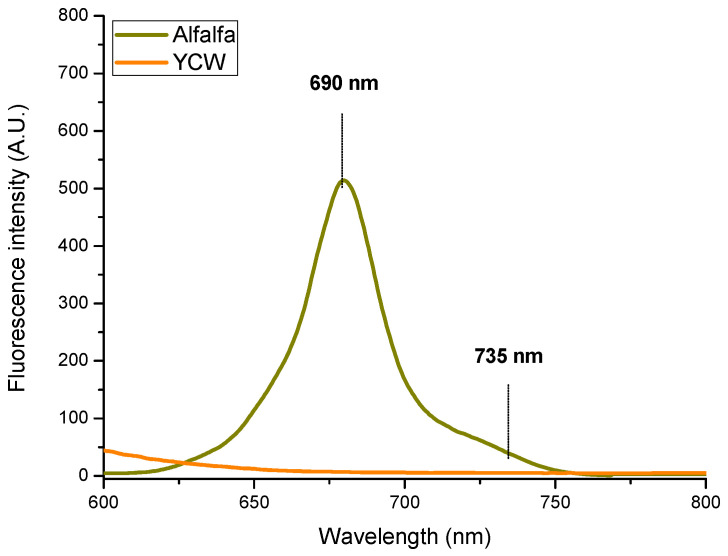
Chlorophyll fluorescence spectra of the adsorbent materials.

Furthermore, Figure 10 shows the fluorescence spectrum of the chlorophylls of the adsorbent materials. The spectrum of the adsorbent prepared from alfalfa leaves shows two fluorescence maximums, one at 690 nm and the other at 735 nm, which correspond to the red and far-red chlorophyll fluorescence, respectively [27,53]. The fluorescence ratio of these two bands F_690_/F_735_ was 8.3. It has been reported that this ratio is a useful tool to detect variations in chlorophyll content in plants and this ratio decreases with increasing the chlorophyll content when re-absorption processes are excluded [27]. Buschmann [54] reported that the F_690_/F_735_ ratio of a diluted leaf extract was 5.7 and this ratio decreased to 0.37 with increases in the chlorophyll concentration (from 3 to 159 μg/mL). In this research, in the fluorescence spectrum of the YCW ethanolic extract, it is clearly observed that this adsorbent material does not contain any pigments.

## 3. The Proposed Adsorption Mechanism 

Adsorbent materials are capable of removing AFB_1_ through a combination of chemical (redox reactions, complex formation, covalent bonds, and proton displacement) and physical (hydrophobic interactions, π–π stacking, dipole–dipole interactions, hydrogen bonding, and Van der Waals forces) mechanisms [55]. These mechanisms can be divided into electrostatic (ionic attractions) and non-electrostatic (dipole–dipole, hydrogen bonding, and hydrophobic) interactions [29]. Electrostatic interactions depend on the pH of the solution; therefore, in the present study, ionic attractions play a minor role during AFB_1_ adsorption. On the other hand, due to the significant presence of hydroxyl, amino, and amide groups, the adsorbent material derived from alfalfa leaves would construct numerous hydrogen bonding networks with the oxygen atoms of the ether, carbonyl, and methoxy groups of the AFB_1_ molecule. Consequently, we propose that this interaction is one of the main mechanisms for AFB_1_ adsorption. In addition, the considerable presence of hydrophobic groups such as methyl and aromatics in the adsorbent derived from alfalfa leaves was favorable for the formation of hydrophobic, dipole–dipole, and π–π stacking interactions [13,27]. Finally, it has been previously described—by our research group—that chlorophyll, which is a highly hydrophobic molecule, can form strong non-covalent complexes with the AFB_1_ molecule, regardless of pH [31]. Therefore, the formation of these complexes also contributed to the AFB_1_ adsorption. The set of these electrostatic and non-electrostatic interactions makes the adsorbent derived from alfalfa leaves an effective material for the adsorption of AFB_1_ from the contaminated feed destined for poultry.

## 4. Conclusions

Taken together, these findings suggest that the use of an adsorbent material derived from alfalfa leaves is a viable alternative for removing AFB_1_ in in vitro digestion models that closely mimic the complex physiological conditions of birds. Both in vitro trials demonstrated that the plant-based adsorbent was efficient for the adsorption of AFB_1_ due to the combination of electrostatic and non-electrostatic interactions. Therefore, this material can be used at a low inclusion level (0.5% *w*/*w*), to successfully remove the AFB_1_ present in poultry feed. However, since in vitro models do not perfectly mimic the GIT conditions, in vivo studies are also required to help determine the efficacy of the alfalfa adsorbent in reducing the toxic effects of AFB_1_. Our group of researchers is already conducting these relevant studies. The results will be published elsewhere.

## 5. Materials and Methods

### 5.1. Chemicals and Reagents

The AFB_1_ standard (CAS number: 1162-65-8), dimethyl sulfoxide (≥99.9% purity, CAS number 67-68-5), HPLC grade methanol (CAS number 67-56-1), 96% ethanol (CAS number 64-17-5), sodium hydroxide (NaOH; ≥97% purity; CAS number 1310-73-2), hydrochloric acid (HCl; ~37% purity; CAS number 7647-01-0), and sodium hypochlorite solution (CAS number 7681-52-9) were acquired from Merck KGaA (Darmstadt, Germany). 

### 5.2. Adsorbent Materials 

The in vitro experiments were carried out with alfalfa leaves (*Medicago sativa* L.), collected in the Botanic Garden of the Superior Studies Faculty at Cuautitlan (National Autonomous University of Mexico). A commercial premium yeast cell wall (YCW) from *Saccharomyces cerevisiae* (SafMannan, Phileo Lesaffre Animal Care, Lesaffre Iberica S.A., Valladolid, Spain) was used as a reference material. Briefly, alfalfa leaves were separated manually and thoroughly washed with distilled water to remove surface-adhered dirt particles. Subsequently, the fresh leaves were dried for 24 h at a constant temperature (40 °C) in an oven (Binder model RE-115, Tuttlingen, Germany). The dried leaves were ground in a C-11-1 type electric plate mill (Glen Mills Inc., Clifton, NJ, USA). Since adsorption is affected by varying the particle size, the ground material was sieved to obtain a size within the optimal range for AFB_1_ adsorption, attaining an average size of <250 µm (60 mesh sieve) as recommended by Zavala-Franco et al. [10]. Finally, the freshly prepared material was stored in a vacuum-sealed plastic container and kept in a desiccator until use. In the in vitro experiments, an inclusion level of 0.5% (*w*/*w*) was utilized, which is in accordance with the safe limit that the Panel on Additives and Products or Substances used in Animal Feed (FEEDAP) considers for a mycotoxin binder montmorillonite [11].

### 5.3. In Vitro Adsorption Studies 

#### 5.3.1. Preparation of the Aflatoxin B_1_ (AFB_1_) Solution and the pH-Dependent Model 

A stock solution of AFB_1_ (100 µg/mL, equivalent to 0.32 mM) was prepared by dissolving the toxin in dimethyl sulfoxide (DMSO). The stock solution was stored in the dark at 4 °C until further use. Then, the stock solution was diluted in deionized water adjusted at three pH values (2, 5, and 7) until reaching a final concentration of 250 ng AFB_1_/mL. 

To assess the efficacy of the adsorbent in removing AFB_1_, a single in vitro model was considered. Samples of 25 mg (0.5% *w*/*w*) of the adsorbent materials were accurately weighed and dispersed in glass vials with 5 mL of the solutions containing the AFB_1_. The flasks were incubated in an agitated water bath (Bellco Glass Inc. Vineland, NJ, USA) and carefully homogenized at 120 rpm at a temperature of 40 °C for 2 h. Subsequently, the adsorbent was separated by centrifugation at 7000× *g* (5810 R centrifuge, Eppendorf, Hamburg, Germany) for 7 min, and the supernatant was filtered through a PTFE membrane syringe filter (pore size 0.22 μm) and subjected to ultra-performance liquid chromatography with fluorescence detection (UPLC-FLR). The pH was determined using a glass electrode (Conductronic PC-45, Puebla, Mexico). All determinations were performed in quintuplicate. Control samples (without the addition of adsorbents) were also included in the experiment to confirm the stability of the AFB_1_ molecule in the different pH media under the same incubation conditions.

#### 5.3.2. Preparation of the AFB_1_-Contaminated Diet and the Avian Intestinal Model

A commercial maize-soybean meal poultry diet (Nutricion Tecnica Animal SA de CV, Queretaro, Mexico) containing 26% protein (12.64 MJ/kg metabolizable energy) was contaminated at a content of 250 µg AFB_1_/kg. Levels of AFB_1_, total fumonisins, and deoxynivalenol were determined in the commercial feed using VICAM’s fluorometric tests based on monoclonal antibody-based affinity columns (VICAM Science Technology, Watertown, MA, USA). To check the homogeneity of the aflatoxin-contaminated feed, five random samples were taken, and the presence of AFB_1_ was confirmed using the 991.31 AOAC procedure [56].

An avian intestinal model was used to evaluate the adsorption capacity of the adsorbent materials using the procedure reported by Hernandez-Patlán et al. [57] with minimal modifications. The test was carried out with one reference material (YCW) and one treatment (the adsorbent prepared from alfalfa leaves). The experiment was conducted at 40 °C to emulate the poultry body temperature with constant agitation (19 rpm). In the first stage, 5 g of the AFB_1_-contaminated feed and 0.5% (*w*/*w*) of the adsorbent material was mixed and poured into polypropylene centrifuge tubes (50 mL capacity). To emulate the crop environment, 10 mL of 0.03 M HCl were added, and tubes were incubated for 30 min. The pH value was around 5. After this period, 2.5 mL of 1.5 M HCl and 3000 U of pepsin (Merck KGaA, Darmstadt, Germany) per gram of feed was added to each tube to emulate the proventriculus environment, reaching a pH of 2. Tubes were incubated again for 45 min. Finally, to emulate the intestinal section, 6.84 mg of 8× pancreatin (Merck KGaA, Darmstadt, Germany) in 6.5 mL of 1.0 M NaHCO_3_ was added to the tubes and incubated for another 120 min. The entire avian intestinal model simulation took 195 min. At the end of the assay, the tubes were centrifuged at 7000× *g* for 10 min, and the residual AFB_1_ concentration remaining in the supernatant was quantified by the UPLC-FLR technique. All determinations were also performed in quintuplicate. Control samples (without the addition of adsorbent materials) were also included to know the real concentration of AFB_1_ per tube under the simulated conditions of the gastrointestinal tract of birds.

The percentage of AFB_1_ adsorbed was computed using the following mathematical expression:

(1) $$Adsorption\;\left(\%\right)=\frac{\left(Ci-Cs\right)}{Ci}\times 100$$

where *Ci* is the concentration of AFB_1_ in the control samples (ng/mL), and *Cs* is the concentration of AFB_1_ in the supernatant (ng/mL).

#### 5.3.3. Analysis of Aflatoxin B_1_ (AFB_1_) 

To quantify the AFB_1_ concentration in the supernatants, immunoaffinity columns based on monoclonal antibodies (Afla-B, VICAM Science Technology, Watertown, MA, USA) were used as a clean-up protocol. Subsequently, the purified toxin was analyzed by UPLC-FLR using a Waters ACQUITY H-class System. Briefly, the methanolic extracts collected from the immunoaffinity columns (1 µL) were injected and eluted with a mobile phase of water:methanol:acetonitrile (64:18:18) at a flow rate of 700 μL/min. The AFB_1_ was detected using excitation and emission wavelengths of 365 nm and 435 nm, respectively. The AFB_1_ concentration was estimated using a reference standard (AFB_1_, Merck KGaA, Darmstadt, Germany) with a calibration curve. The detection limit of AFB_1_ was found to be 0.002 µg/L.

### 5.4. Characterization 

#### 5.4.1. Fourier Transform Infrared Spectroscopy with Attenuated Total Reflection (FTIR-ATR) 

Infrared spectra were collected using a Frontier SP8000 Fourier transform infrared spectrophotometer (Perkin Elmer, Waltham, MA, USA) equipped with an attenuated total reflection (ATR) attachment (DuraSamplIR II, Smiths Detection, Warrington, UK). The adsorbents were scanned in the 4000–400 cm^−1^ region. The bond indexes (BIs) of the principal functional groups were calculated using the following mathematical expressions:

(2) $${BI}_{OH}=\frac{{BA}_{3281}}{\sum BA}$$

(3) $${BI}_{\left({CH}_{2}\right)n}=\frac{{BA}_{2916}+{BA}_{2850}}{\sum BA}$$

(4) $${BI}_{COOR}=\frac{{BA}_{1613}}{\sum BA}$$

(5) $${BI}_{C=C}=\frac{{BA}_{1406}}{\sum BA}$$

(6) $${BI}_{C-O}=\frac{{BA}_{1031}}{\sum BA}$$

where BA_x_ is the band area around the corresponding wavenumber (cm^−1^); and ΣBA is the total area of all bands in the corresponding FTIR spectrum.

#### 5.4.2. Environmental Scanning Electron Microscopy (ESEM)

The microstructure and morphology of the adsorbents were characterized by environmental scanning electron microscopy (Philips-XL30 ESEM, Eindhoven, The Netherlands) with an accelerating voltage of 3, 5, and 20 kV. Samples were coated with a thin layer of gold using a sputter coater (Denton Vacuum Inc., Desk V HP, Moorestown, NJ, USA) operated at 7 mA for 3 min. Microscopy analysis (2500× and 5000×) was performed in a secondary electron imaging (SEI) mode. 

#### 5.4.3. X-ray Fluorescence Spectroscopy (XRF)

The multi-elemental analysis was conducted in triplicate, using a high-performance XTrace microspot X-ray source, and the photon-induced micro-X-ray fluorescence spectrum was measured with the XFlash^®^ 6/10 silicon drift detector (Bruker Nano GmbH, Berlin, Germany). The technique was carried out using an environmental scanning electron microscope equipped with X-ray fluorescence spectroscopy (Phillips XL30/40 XRF-ESEM, Eindhoven, The Netherlands).

#### 5.4.4. X-ray Diffraction (XRD)

The X-ray diffraction measurements were conducted on a 2100-Rigaku diffractometer (Rigaku Co., Tokyo, Japan). The diffraction data were recorded for 2θ between 5° and 70° with a resolution of 0.02°.

#### 5.4.5. Point of Zero Charge (pH_pzc_) and Zeta Potential (ζ-Potential)

The point of zero charge (pH_pzc_) was determined following the procedure described by Zavala-Franco et al. [10]. In brief, samples of each of the adsorbent materials (25 mg) were weighed in five flasks containing deionized water adjusted at different pH values (pH_i_=2, 5, 7, 9, and 11). Subsequently, samples were shaken at 200 rpm at room temperature for 195 min. Thereafter, the final pH (pH_f_) of the supernatant was recorded using a glass electrode. A plot of pH_pzc_ was constructed as follows: ΔpH (pH_i_–pH_f_) against pH_i_. On the other hand, Zeta potential (ζ-potential) measurements were performed using the ZetaSizer Pro (Malvern Instruments, Worcestershire, UK) following the recommendations of Ramales-Valderrama [11]. All determinations were conducted at room temperature in quintuplicate. 

#### 5.4.6. Chlorophyll and Carotenoid Quantification 

A Lambda 365 UV-Vis-diffuse reflectance spectrophotometer (Perkin Elmer, Waltham, MA, USA) equipped with a 100-mm integrating sphere was used. The optical absorption spectra of powders were collected in the range of 400–800 nm. The pigments such as chlorophyll *a* (Chl *a*), chlorophyll *b* (Chl *b*), and total carotenoids (xanthophylls + carotenes) were extracted with 96% ethanol and characterized using a Cary 8454 UV-Vis Diode Array System spectrophotometer (Agilent Technologies, Santa Clara, CA, USA). The absorption coefficients reported by Lichtenthaler and Wellburn [51] were utilized for pigment quantification.

### 5.5. Experimental Design and Statistical Analysis

The experiments were conducted as a completely randomized design, and data were analyzed by means of a one-way analysis of variance (ANOVA). The Tukey test was used to compare the means from the ANOVA. The threshold for significance level was set at α = 0.05. The OriginPro v8 software was used.

## Figures and Tables

**Table 1:** Bands assignments and functional groups in the adsorbent material derived from alfalfa leaves and the yeast cell wall (YCW).

Band	Wavenumber (cm^−1^)		Functional Group
	Alfalfa	YCW	
A	3668	NF	O–H stretching
B	3280	3281	O–H and N–H stretching vibrations (carbohydrate and protein)
C	2964	NF	CH_2_ antisymmetric stretching (lipids)
D	2917	2923	–(CH_2_)_n_– antisymmetric stretching (lipids)
E	2850	2853	C–CH_3_ symmetric stretching (lipids)
F	1732	1710	C=O stretching (phospholipid esters)
G	NF	1629	Amide I (N–H bending and C=O stretching)
H	1599	NF	COOR (carboxylate group)
I	NF	1532	Amide II (C–N stretching and N–H bending)
J	NF	1455	OH bending vibration in carboxylic acids
K	1408	NF	–CH_2_ deformation (cellulose)
L	NF	1369	β-anomeric carbons (β-glucans)
M	1242	1244	PO_2_^−^ antisymmetric stretching (DNA, RNA, phospholipid, phosphorylated protein)
N	1066	NF	C–O stretching (carbohydrate)jialiC–O–P stretching (phosphate ester)
O	NF	1025	C–O stretching (carbohydrates)
P	NF	887	β-anomeric carbons β (1→3)-glucans
Q	NF	812	Mannans (C–O–C, C–C, and C–OH stretching of pyranose ring)
R	NF	670	Polysaccharides (α- and β-glucans, α-mannan)
S	611	NF	NH_2_ wag (primary amines)
T	NF	575	Polysaccharides (α- and β-glucans, α-mannan)
U	534	NF	In plane and out-of-plane ring deformations
V	NF	508	Polysaccharides (α- and β-glucans, α-mannan)

YCW = yeast cell wall. NF = not found.

**Table 2:** Chlorophylls and total carotenoid contents of the adsorbent materials.

Photosynthetic Pigment	Content (µg/g Dry Weight)	
	Alfalfa	YCW
Chlorophyll *a*	1251.2.1 ± 84.4	ND
Chlorophyll *b*	1508.0 ± 132.8	ND
Total chlorophyll (*a + b*)	2759.1 ± 180.2	ND
Total carotenoid (*x* + *c*)	16.3 ± 94.5	ND

x + c = xantophyll + carotenes. ND = not detected.

## Data Availability

The datasets used and or analyzed during the current study are available from the corresponding author upon reasonable request.

## Supplementary Materials

The following supporting information can be downloaded at: https://www.mdpi.com/article/10.3390/toxins15100604/s1, Figure S1: UV-Vis spectrum of the main pigments contained in the adsorbent materials.
