# Supplementary material for: Removal of Aflatoxin B1 Using Alfalfa Leaves as an Adsorbent Material: A Comparison between Two In Vitro Experimental Models
Source: Toxins (Basel). 2023 Oct 8;15(10):604. doi: 10.3390/toxins15100604 (PMC10610884; doi:10.3390/toxins15100604)
Supplement: Supplementary file 1 [file toxins-15-00604-s001.zip › toxins-2629148-supplementary.docx]

Supplementary Materials: Removal of Aflatoxin B_1_ Using
Alfalfa Leaves as an Adsorbent Material: A Comparison
between Two In Vitro Experimental Models

María de Jesús Nava-Ramírez, Alma Vázquez-Durán, Juan de Dios Figueroa-Cárdenas, Daniel Hernández-Patlán, Bruno Solís-Cruz, Guillermo Téllez-Isaías, Carlos López-Coello and Abraham Méndez-Albores

**Figure S1.** UV-Vis spectrum of the main pigments contained in the adsorbent materials.
